# Improving risk stratification of patients with childhood acute lymphoblastic leukemia: Glutathione-S-Transferases polymorphisms are associated with increased risk of relapse

**DOI:** 10.18632/oncotarget.8606

**Published:** 2016-04-06

**Authors:** Daiana B. Leonardi, Mercedes Abbate, María C. Riccheri, Myriam Nuñez, Graciela Alfonso, Geraldine Gueron, Adriana De Siervi, Elba Vazquez, Javier Cotignola

**Affiliations:** ^1^ Laboratorio de Inflamación y Cáncer, Departamento de Química Biológica, Facultad de Ciencias Exactas y Naturales-Universidad de Buenos Aires, IQUIBICEN-CONICET, Intendente Güiraldes 2160 (1428), CABA, Argentina; ^2^ Departamento de Hematología Pediátrica, Hospital Nacional Profesor A. Posadas, Pte. Illia s/n (1684), El Palomar, Buenos Aires, Argentina; ^3^ Departamento de Matemáticas, Facultad de Farmacia y Bioquímica, Universidad de Buenos Aires, Junín 954 (1113), CABA, Argentina; ^4^ Departamento de Hematología, Hospital Nacional Profesor A. Posadas, Pte. Illia s/n (1684), El Palomar, Buenos Aires, Argentina; ^5^ Current Affiliation: Laboratorio de Oncología Molecular y Nuevos Blancos Terapéuticos, Instituto de Biología y Medicina Experimental (IBYME–CONICET), Vuelta de Obligado 2490 (1428), CABA, Argentina

**Keywords:** acute leukemia, Glutathione-S-Transferase, polymorphism, predictor, relapse

## Abstract

The inclusion of genotype at Acute Lymphoblastic Leukemia (ALL) diagnosis as a genetic predictor of disease outcome is under constant study. However, results are inconclusive and seem to be population specific. We analyzed the predictive value of germline polymorphisms for childhood ALL relapse and survival. We retrospectively recruited 140 Argentine patients with *de novo* ALL. Genotypes were analyzed using PCR-RFLP (*GSTP1* c.313A > G, *MDR1* c.3435T > C, and *MTHFR* c.665C > T) and multiplex PCR (*GSTT1* null, *GSTM1* null). Patients with the *GSTP1* c.313GG genotype had an increased risk for relapse in univariate (OR = 2.65, 95% CI = 1.03–6.82, *p* = 0.04) and multivariate (OR = 3.22, 95% CI = 1.17–8.83, *p* = 0.02) models. The combined genotype slightly increased risk for relapse in the univariate (OR = 2.82, 95% CI = 1.09–7.32, *p* = 0.03) and multivariate (OR = 2.98, 95% CI = 1.14–7.79, *p* = 0.03) models for patients with 2/3-risk-genotypes (*GSTT1* null, *GSTM1* null, *GSTP1* c.313GG). The Recurrence-Free Survival (RFS) was shorter for *GSTP1* c.313GG (*p* = 0.025) and 2/3-risk-genotypes (*p* = 0.021). GST polymorphisms increased the risk of relapse and RFS of patients with childhood ALL. The inclusion of these genetic markers in ALL treatment protocols might improve risk stratification and reduce the number of relapses and deaths.

## INTRODUCTION

Current treatment protocols of the International Berlin-Frankfurt-Münster Study Group (I-BFM-SG) for childhood Acute Lymphoblastic Leukemia (ALL) include the stratification of patients into groups of risk for disease relapse. This stratification is based on biochemical and cytogenetic parameters at diagnosis, and the early response to treatment determined by the Minimal Residual Disease (MRD). BFM current protocols also indicate that patients may be re-assigned to another group during induction according to their MRD at different days (e.g. day 15). Chemotherapy protocols for ALL are complex and can last two to three years. Each risk group (standard, intermediate and high) is treated with specific chemotherapy schemas to reduce to a minimum the number of relapses, chemotherapy toxicity and treatment-related deaths.

The inclusion of genotype as an additional molecular risk factor for relapse or drug toxicity is a promising tool to further increase survival and improve quality of life. Several studies have been conducted to analyze genetic variants as predictors of disease outcome; however, results are still contradictory and inconclusive, and seem to be population specific due to the multiple factors involved in tumor development and progression [1].

Previous reports demonstrated that a Single Nucleotide Polymorphism (SNP) in *MTHFR* increases the risk of dying of ALL among adults and the risk of hepatotoxicity [2]. A genomic study performed on patients with childhood ALL from the St. Jude Total Therapy and the Children's Oncology Group (COG) protocols found that several SNPs correlated with early response to treatment, disease relapse or altered drug metabolism [3]. Another study that included adult patients with Acute Myeloid Leukemia showed the *GSTM1* null genotype shortened the disease-free survival [4]; and this association was even stronger when the *GSTT1* and *GSTM1* null genotypes were combined [4].

Altogether, these and other reports support the need to undertake more molecular studies to validate additional risk factors that allow optimizing stratification protocols and, in consequence, increase survival rates. Here, we analyzed five polymorphisms in enzymes participating in key pathways involved in acute leukemia development, progression and targeted for therapy. The enzyme MTHFR participates in folate and methotrexate metabolism and the c.665C > T SNP modifies the enzymatic activity [5]. ABCB1/MDR1 is an efflux bomb for a wide variety of xenobiotics and is frequently responsible for the development of resistance to antineoplastic drugs and the polymorphism *MDR1* c.3435T > C alters gene expression levels [6]. GSTs are a family of detoxifying enzymes; GSTP1 c.313A > G SNP modifies the enzyme activity [7], and the genes encoding for GSTT1 and GSTM1 are frequently deleted.

Currently, no studies have been performed in the Argentine population to establish the association between polymorphisms and ALL relapse. Therefore, we sought to study germline variants and their predictive value for disease relapse in pediatric patients with ALL.

## RESULTS

### Analysis of single polymorphisms

**Figure 1:**
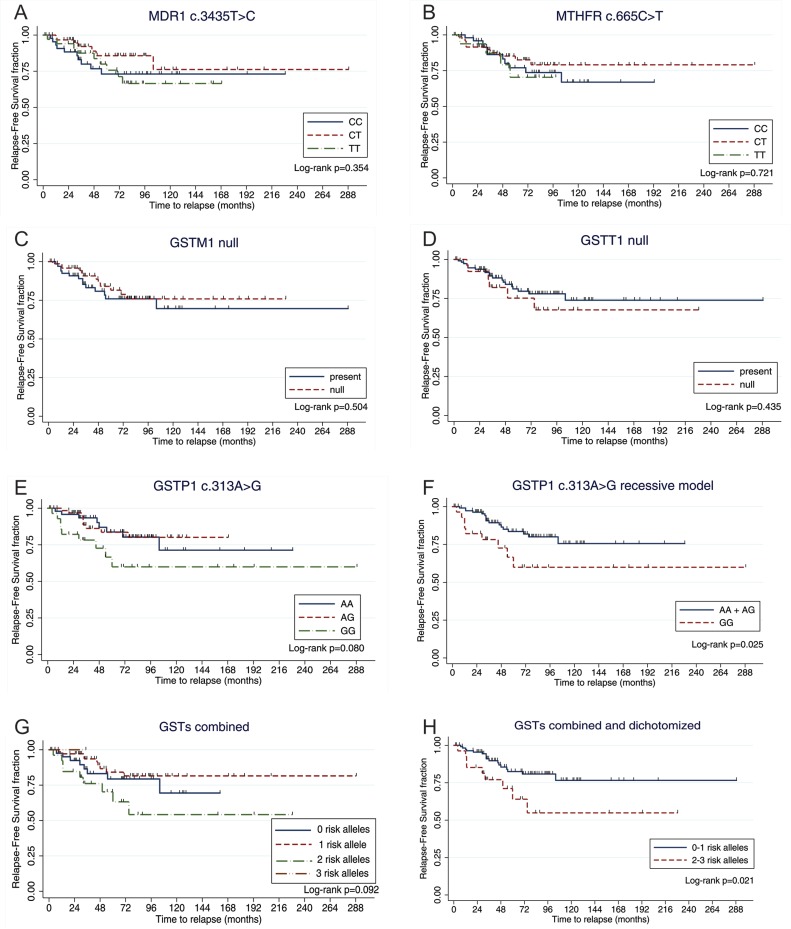
Analyses of Relapse-Free survival stratified by genotype The figure depicts the Kaplan–Meier survival analysis by genotype. Time was calculated from date of diagnosis to date of disease recurrence or last follow-up. Marks denote censored patients. The *GSTP1* c.313GG genotype shortened the RFS when the recessive model was considered. Higher number of GSTs risk alleles were also associated with poorer RFS.

To study the association between the polymorphisms and relapse, we first tested an additive genetic model, where the heterozygote genotypes show intermediate enzyme activity and the homozygotes wild-type and variant have the highest and lowest activities, respectively. Non-statistical significant associations were found (Table 1). Similarly, the analyses of Recurrence-Free Survival (RFS) showed non-significant differences between the individual genotypes when the additive model was used (Figure 1A–1E).

**Table 1:** Analyses of association between genotypes and relapse

		RELAPSE		*p*-val^a^	OR (95% CI)	*p*-val
		NO	YES			
**MDR c.3435**	CC	35 (77.8%)	10 (22.2%)		1 (reference)	
	CT	53 (88.3%)	7 (11.7%)		0.46 (0.16–1.33)	0.152
	TT	25 (73.5%)	9 (26.5%)	0.160	1.26 (0.45–3.55)	0.662
**MTHFR c.665**	CC	40 (78.4%)	11 (21.6%)		1 (reference)	
	CT	60 (84.5%)	11 (15.5%)		0.67 (0.26–1.68)	0.391
	TT	13 (76.5%)	4 (23.5%)	0.601	1.12 (0.30–4.12)	0.866
**GSTP1 c.313**	AA	39 (83.0%)	8 (17.0%)		1 (reference)	
	AG	56 (86.1%)	9 (13.9%)		0.78 (0.27–2.21)	0.644
	GG	19 (67.9%)	9 (32.1%)	0.108	2.31 (0.77–6.93)	0.136
**GSTP1 c.313 recessive**	AA + AG	95 (84.8%)	17 (15.2%)		1 (reference)	
	GG	19 (67.9%)	9 (32.1%)	**0.039**	2.65 (1.03–6.82)	**0.044**
**GSTM1**	PRESENT	52 (78.8%)	14 (21.2%)		1 (reference)	
	NULL	62 (83.8%)	12 (16.2%)	0.448	0.72 (0.31–1.69)	0.449
**GSTT1**	PRESENT	95 (82.6%)	20 (17.4%)		1 (reference)	
	NULL	19 (76.0%)	6 (24.0%)	0.441	1.50 (0.53–4.23)	0.443
**GSTs**	0	33 (80.5%)	8 (19.5%)		1 (reference)	
	1	63 (87.5%)	9 (12.5%)		0.59 (0.21–1.67)	0.320
	2	17 (65.4%)	9 (34.6%)		2.18 (0.72–6.68)	0.171
	3	1 (100.0%)	0 (0.0%)	0.092	nd^b^	
**GSTs**	0–1	96 (85.0%)	17 (15.0%)		1 (reference)	
	2–3	18 (66.7%)	9 (33.3%)	**0.028**	2.82 (1.09–7.32)	**0.033**

a chi-square *p*-values for the analysis of association between genotype and relapse.

b nd: cannot be determined.

Statistical significant *p*-values are bolded.

Given that meaningful differences were not observed between the survival of patients with the *GSTP1* c.313AA and c.313AG genotypes (Figure 1E), we tested a recessive model in which a reduction of enzyme activity would be only observed on the homozygote G genotype. We found a significant association between the *GSTP1* c.313GG genotype and relapse (*p* = 0.039), and an increased risk for recurrence (OR = 2.65, 95% CI = 1.03–6.82, *p* = 0.044; Table 1). The higher risk for relapse remained significant when the model was adjusted for the other polymorphisms (ORadj = 3.22, 95% CI = 1.17–8.83, *p* = 0.023). This genotype also shorten RFS (Figure 1F and Table 2).

**Table 2:** Estimation of Hazard Ratios for relapse according to genotype

		HR	95% CI	adj. *p*-value*
**GSTP1 c.313 recessive**	AA + AG	1.00	Reference	
	GG	2.58	1.10–6.06	0.030
**GSTs**	0–1 risk alleles	1.00	Reference	
	2–3 risk alleles	2.50	1.11–5.62	0.027

* adjusted for gender, age at diagnosis, risk group, treatment protocol and genotype.

### Analysis of polymorphisms combinations

Since GSTs are enzymes that participate in the same biological pathways with overlapping substrate specificity, we considered an additive score that captures information on the genotypes of *GSTT1*, *GSTM1* and *GSTP1*. We stratified the genotypes as follows: 0-risk-allele genotype (*GSTT1* present, *GSTM1* present, and *GSTP1* c.313AA/AG), 1-risk-allele genotype (*GSTT1* null, *GSTM1* present, and *GSTP1* c.313AA/AG; or *GSTT1* present, *GSTM1* null, and *GSTP1* c.313AA/AG; or *GSTT1* present, *GSTM1* present, and *GSTP1* c.313GG), 2-risk-allele genotype (*GSTT1* null, *GSTM1* null, and *GSTP1* c.313AA/AG; or *GSTT1* null, *GSTM1* present, and *GSTP1* c.313GG; or *GSTT1* present,*GSTM1* null, and *GSTP1* c.313GG), and 3-risk-allele genotype (*GSTT1* null, *GSTM1* null, and *GSTP1* c.313GG). Non-statistical significant associations were observed when the additive model was tested (Figure 1G and Table 1).

Since patients within the 0- and 1-risk-allele groups did not show significant differences in RFS (Figure 1G) and because there was only one patient with three risk-alleles, we dichotomized this variable into low-risk (0/1 risk alleles) and high-risk (2/3 risk alleles) genotypes. The high-risk genotype was associated with a nearly 3-fold increased risk for disease recurrence in the univariate model (Table 1) and when adjusted for the other genotypes (ORadj = 2.98, 95% CI = 1.14–7.79, *p* = 0.026). Patients with the high-risk genotype also had shorter RFS (Figure 1H and Table 2).

## DISCUSSION

Identifying associations between genetic factors and disease outcome is very important for epidemiological research and for improving survival and quality of life. This study, performed using samples derived from Argentine patients with Acute Leukemia, revealed that a variant allele of *GSTP1* increased the risk of childhood ALL relapse and shortened the RFS. The association was stronger when the combined genotype of *GSTP1*, *GSTT1* and *GSTM1* was considered.

Current chemotherapy for ALL involves complex multidrug protocols and requires a thorough follow up to reduce relapses, therapy-related toxicities and deaths. The inclusion of the genetic background into clinical protocols might help to optimize the stratification and to comprehensively monitor the treatment. Therefore, there is a need to undertake more studies to find and validate predictive markers, especially in understudied populations.

Here, we analyzed polymorphisms in enzymes participating in key pathways involved in acute leukemia development, progression and targeted for therapy. We did not find statistical significant associations between *MTHFR* c.665C > T SNP and RFS, probably due to the moderate number of samples studied or by the effect of other common polymorphism (NM_005957.4:c.1286A > C; formerly c.1298A > C) not included in this protocol. There is only one study in Argentina that analyzed MTHFR c.665C > T SNP on patients with childhood ALL who received methotrexate. The study reported that the T allele increased risk of leukopenia and neutropenia [8]. Similarly, other studies found that homozygote T patients were at higher risk for disease relapse [9], had increased risk of toxicity [2, 10, 11], and presented worse survival [2, 10]. On the other hand, it was reported that the c.665TT genotype increased the overall survival in Brazilian patients with childhood ALL [12].

We did not find significant associations between ABCB1/MDR1 polymorphism and ALL outcome. Similar results were found by others [13]; however, some studies showed that homozygote C patients with AL had worse survival [14–16].

We also observed that the *GSTP1* c.313A > G polymorphism increased the risk for relapse and shortened the RFS in homozygote G patients. The combined genotype of the three GSTs revealed that patients with 2/3 risk-allele genotypes had higher risk of relapse and worse RFS. Similarly, Voso *et al.* reported that patients with a less detoxifying genotype (*GSTT1* and *GSTM1* double null genotype) had worse overall survival [17]. In contrast, other groups showed that children with ALL and *GSTT1* null or *GSTM1* null or *GSTP1* c.313AA genotypes had lower risk for relapse [18, 19]. Comparable results were also published for AML and childhood ALL [4, 20, 21].

Interestingly, we previously published that GST polymorphisms affect prostate cancer RFS [22], which indicate that these polymorphisms might be relevant predictors for cancer outcome.

There are several possible reasons for the inconsistencies between studies; for example, the small to moderate population sizes, different treatment protocols, different ethnic/genetic backgrounds, and other cofounders such as environmental factors. Key limitations for our study are the modest number of patients included, and that some patients were followed for periods shorter than the median time to relapse.

In conclusion, GST polymorphisms that reduce or eliminate enzyme activity increased the risk of relapse and shortened the RFS in pediatric patients with ALL. These data warrant the validation of results on a larger patient cohort and the inclusion of genetic markers into the clinic in an effort to improve risk stratification childhood ALL.

## MATERIALS AND METHODS

### Patients

We designed a hospital-based case study to find predictors of childhood ALL relapse. We retrospectively recruited patients diagnosed with *de novo* ALL from August 2007 to July 2013 at the *Hospital Nacional Profesor Alejandro Posadas*, Buenos Aires, Argentina. The protocol was approved by the Institutional Ethical Committee in compliance with the Ethical Principles enunciated by the Declaration of Helsinki. Patients were considered pediatric when they were diagnosed with ALL at an age younger than 20 years old, and infants when the age at diagnosis was < 1 year old. All patients who agreed to participate in the study, and their legal guardians, signed a written informed consent prior sample donation.

Patient recruitment, follow-up and maintenance of updated medical records were performed by trained oncohematologists. Patients were treated according to BFM-based protocols. The study group consisted of 140 pediatric patients diagnosed with ALL who achieved complete remission during treatment. The clinico-pathological characteristics and genotype frequencies are shown in Table 3.

**Table 3:** Clinico-pathologic characteristics

Total *n* Clinical features	140^a^
**Age at diagnosis**	
Average (years old)	6
Median (years old)	5
Range (years old)	0–19
**Follow-up time**	
Average (months)	65
Median (months)	53
Range (months)	2–288
**Gender**	
Males	66 (47%)
Females	74 (53%)
**Risk group^b^**	
Standard	47 (34%)
Intermediate	73 (54%)
High	16 (12%)
Missing data	4
**Relapse**	
No	114 (81%)
Yes	26 (19%)
**Time to relapse**	
Average (months)	36
Median (months)	33
Range (months)	4–104
**GENOTYPES *MDR* c.3435**	
CC	45 (32%)
CT	60 (43%)
TT	34 (25%)
Missing	1
***MTHFR* c.665**	
CC	51 (37%)
CT	71 (51%)
TT	17 (12%)
Missing	1
***GSTP1*** **c.313**	
AA	47 (34%)
AG	65 (46%)
GG	28 (20%)
Missing	0
***GSTT1***	
Present	115 (82%)
Null	25 (18%)
Missing	0
***GSTM1***	
Present	66 (47%)
Null	74 (32%)
Missing	0

a includes 3 infant cases (< 1 year old).

b stratification according to BFM-GATLA protocols.

All patients were Argentine citizens, and by definition Hispanics. Most of them had predominant Caucasian ancestry, although as reported for this population, some admixture of Amerindian and African ancestry is to be expected [23].

### Genotyping

Germline DNA was extracted from peripheral blood anti-coagulated with EDTA during complete remission of disease or after treatment completion. We genotyped five polymorphisms: *GSTP1* NM_000852.3:c.313A > G (p.Ile105Val; rs1695), *GSTT1* null, *GSTM1* null, *ABCB1/MDR1* NM_000927.3:c.3435T > C (p.Ile1145=; rs1045642), and*MTHFR* NM_005957.4:c.665C > T (p.Ala222Val; rs1801133; formerly called c.677C > T).

The genotyping of *GSTP1* c.313A > G, *ABCB1/MDR1* c.3435T > C and *MTHFR* c.665C > T were performed by PCR-RFLP assays using *Alw26I*, *Bsp143I* or *HinfI* restriction enzymes (Fermentas, Pittsburgh PA, USA), respectively. All enzymatic digestions were performed at 37°C overnight following the manufacturer recommendations.*GSTT1* null and *GSTM1* null genotypes were assessed by multiplex-PCR reaction. This method allowed us to discriminate the null genotype (homozygote deletion) from the heterozygote and homozygote present genotypes. We called the null genotype for either *GSTT1* or *GSTM1* when the specific band was absent and with the specific band for the other gene being present (PCR internal control). Samples that did not amplify for both genes were repeated as needed to discard a PCR failure. These samples were called null for both *GSTT1* and *GSTM1* only when the following criteria were met: i) all replicates were concordant, ii) other samples within the same PCR reaction using the same PCR mix amplified (reaction control), and iii) PCR reactions for double-null samples showed the specific amplicon for other genes (DNA quality control). Details of methods are available as Supplementary Table S1.

All single nucleotide polymorphisms (SNPs) were in Hardy-Weinberg Equilibrium. Genotyping call rates were: 100% for *GSTP1*, 100% for *GSTT1*, 100% for *GSTM1*, 99% for *ABCB1/MDR1*, and 99% for *MTHFR*. All PCR reactions were performed in a DNA Engine™ Thermocycler (Bio-rad, California, USA). PCR reactions and digested products were analyzed by 1.5–2% agarose (Genbiotech SRL, Buenos Aires, Argentina) gel electrophoresis in 1x TAE buffer (0.8 M Tris; 0.4 M sodium acetate; 0.04 M EDTA; pH 8.3) and dyed with ethidium bromide (Promega, Wisconsin, USA). Gels were photographed and analyzed with the G-Box system (Syngene, USA) and the Genesnap software (Syngene, USA).

Samples that failed to amplify or showed non-conclusive genotypes were repeated once or twice as needed. Genotyping outputs were read by two independent laboratory members, and 10–12% of blindly random selected samples were re-analyzed as quality control of the experiments. The results were considered for the final analyses when there was 100% agreement between the two members, and when there was a 100% concordance between samples and blinded repeats.

### Statistical analysis

We performed Chi-square tests to study the association between the genotypes and disease relapse. Logistic regression was used to calculate the Odds Ratios (OR) and 95% Confidence Intervals (95% CI) for disease relapse. We performed Kaplan-Meier plots to evaluate the association between genotypes and Relapse-Free Survival (RFS). Time to relapse was calculated from date of diagnosis to date of relapse or last follow up (censored patients), and the comparison between groups was done using the Log-rank test. Multivariate analyses were conducted using Cox proportional hazard models to study the association between polymorphisms and time to relapse, and to estimate the Hazard Ratios (HR) and 95% CI. Multivariate models included gender, risk group, treatment protocol, age at diagnosis and genotypes as covariates. Differences between groups were considered significant when *p*-value ≤ 0.05. The investigators were blinded to all clinico-pathological variables at the time of genotyping. All statistical analyses were carried out using IBM SPSS Software (IBM Company).

## SUPPLEMENTARY MATERIALS




