# Supplementary material for: Improving risk stratification of patients with childhood acute lymphoblastic leukemia: Glutathione-S-Transferases polymorphisms are associated with increased risk of relapse
Source: Oncotarget. 2016 Apr 6;8(1):110–7. doi: 10.18632/oncotarget.8606 (PMC5352038; doi:10.18632/oncotarget.8606)
Supplement: Supplementary file 1 [file oncotarget-08-110-s001.pdf]

Improving risk stratification of patients with childhood acute lymphoblastic leukemia: Glutathione-S-Transferases polymorphisms are associated with increased risk of relapse

Supplementary Materials

Supplementary Table S1: Table with detailed information for PCR primer sequences and reaction conditions
